# Designing intelligent chatbots with ChatGPT: a framework for development and implementation

**DOI:** 10.3389/frai.2025.1618791

**Published:** 2026-01-05

**Authors:** Sajjad Hyder, Javeed Kittur

**Affiliations:** 1 Biomedical Engineering, The University of Oklahoma, Norman, OK, United States; 2 Engineering Pathways, The University of Oklahoma, Norman, OK, United States

**Keywords:** chatbot, ChatGPT, user experience, prompt, hybrid design model, systematic literature review

## Abstract

**Background:**

The rapid evolution of interactive AI has reshaped human-computer interaction, with ChatGPT emerging as a key tool for chatbot development. Industries such as healthcare, customer service, and education increasingly integrate chatbots, highlighting the need for a structured development framework.

**Purpose:**

This study proposes a framework for designing intelligent chatbots using ChatGPT, focusing on user experience, hybrid design models, prompt engineering, and system limitations. The framework aims to bridge the gap between technical innovation and real-world application.

**Methods:**

A systematic literature review (SLR) was conducted, analyzing 40 relevant studies. The research was structured around three key questions: (1) How do user experience and engagement influence chatbot performance? (2) How do hybrid design models improve chatbot performance? (3) What are the limitations of using ChatGPT, and how does prompt engineering affect responses?

**Results:**

The findings emphasize that well-designed user interactions enhance engagement and trust. Hybrid models integrating rule-based and machine learning techniques improve chatbot functionality. However, challenges such as response inconsistencies, ethical concerns, and prompt sensitivity require careful consideration. A framework for design, development, and implementation of effective Chatbots with ChatGPT has been proposed in this study.

**Conclusion:**

This study provides a structured framework for chatbot development with ChatGPT, offering insights into optimizing user experience, leveraging hybrid design, and mitigating limitations. The proposed framework serves as a practical guide for researchers, developers, and businesses aiming to create intelligent, user-centric chatbot solutions.

## Introduction

The emergence of advanced interactive AI systems has redefined how humans interact with technology, with ChatGPT standing out as a transformative tool in the development of intelligent chatbots. Powered by OpenAI’s GPT architecture, ChatGPT leverages large-scale language models based on the transformer framework to facilitate nuanced, context-sensitive conversations, making it a versatile solution for diverse applications (Tucudean et al., 2024). The GPT architecture utilizes a decoder-only transformer with unidirectional processing, enabling effective text generation and context understanding across multiple domains including healthcare, education, and customer service (Liu, 2024). This study focuses specifically on ChatGPT rather than other large language models (LLMs) such as Gemini, Perplexity, Copilot, or open-source alternatives like Llama, Mistral, and Qwen for several reasons. First, ChatGPT has achieved the widest adoption in enterprise and educational settings, making it the most practically relevant for framework development (Yenduri et al., 2023). Second, ChatGPT’s extensive API ecosystem and integration capabilities provide the most comprehensive development environment for chatbot implementation (Yenduri et al., 2023). Third, the majority of existing literature on conversational AI development has focused on ChatGPT-based implementations, allowing for more robust synthesis and comparison of findings (Yenduri et al., 2023). Industries such as healthcare, customer service, and education are increasingly incorporating chatbots to enhance user engagement, streamline operations, and improve accessibility (Bettayeb et al., 2024; Li et al., 2024; Tan et al., 2024). Despite its widespread use, a clear framework is still needed to guide the effective development and implementation of ChatGPT-based chatbots (Tan et al., 2024).

This research addresses that need by conducting a systematic literature review (SLR) of recent studies published between 2020 and 2025. It aims to explore how user experience, hybrid design models, and prompt engineering influence chatbot performance and effectiveness. Specifically, this review investigates three key questions: (1) How do user experience and engagement influence chatbot performance? (2) How can hybrid design models improve chatbot performance? (3) What are the limitations of using ChatGPT, and how does prompt engineering affect responses?

By synthesizing the findings of 40 publications, this paper offers a comprehensive framework that connects concepts between technical innovation and practical implementation. It provides actionable insights for researchers, developers, and business leaders seeking to harness ChatGPT’s capabilities to create intelligent, content dense, and user-focused chatbot solutions. This work contributes to the evolving discourse on chatbot development, offering both theoretical foundations and practical guidelines to support future advancements in the field.

## Related work

While several systematic reviews have examined conversational AI systems broadly, limited research has specifically focused on ChatGPT-based chatbot development frameworks. Recent surveys by Casheekar et al. (2024) examined AI-powered conversational agents with emphasis on UI/UX considerations, while Cerny (2023) provided an overview of educational chatbot applications. However, these studies lack a comprehensive, implementable framework that specifically addresses the unique characteristics of ChatGPT integration, hybrid design approaches, and prompt engineering strategies. The following Table 1 represents a comparison among Casheekar et al. (2024), Cerny (2023), and our own manuscript to showcase the contrasts and notable differences.

**Table 1:** Frameworks of intelligent chatbot design.

Aspect	Casheekar et al. (2024)	Cerny (2023)	Our study
Scope	General AI-powered conversational agents	Educational chatbots (broad)	Specifically ChatGPT-based chatbots
Framework	Broad UI/UX guidelines	Pedagogical considerations	Detailed 3-phase implementation framework
Technical Depth	Limited implementation details	Conceptual overview	Comprehensive technical guidance
Hybrid Models	Not addressed	Brief mention	Detailed analysis with exemplar studies
Methodology	Narrative review	Qualitative analysis	PRISMA-guided systematic review

## Methods

The PRISMA framework was utilized in this research to guide the SLR process (Page et al., 2021). This framework has three main phases: Phase 1 identification: as articles are retrieved from different databases using specific search terms. Phase 2 screening: as articles are screened by their abstract and full text which follows a preset exclusion criterion. Phase 3 inclusion: as the final articles included in the review process are analyzed in detail to address a set of research questions. The PRISMA framework was selected to guide the SLR process for its transparency and methodological rigor. The SLR process began by entering different search terms into several databases (Borrego et al., 2014; Clapton et al., 2009; James et al., 2016). The term “Chatbot design + ChatGPT” and related terms were used as a search term/phrase in this study to retrieve articles. The databases used to find articles were Google Scholar, IEEE Xplore Digital Library, ERIC (Education Resources Information Center), Science Direct, Compendex, and Wiley Online Library.

### Data collection

**Figure 1:**
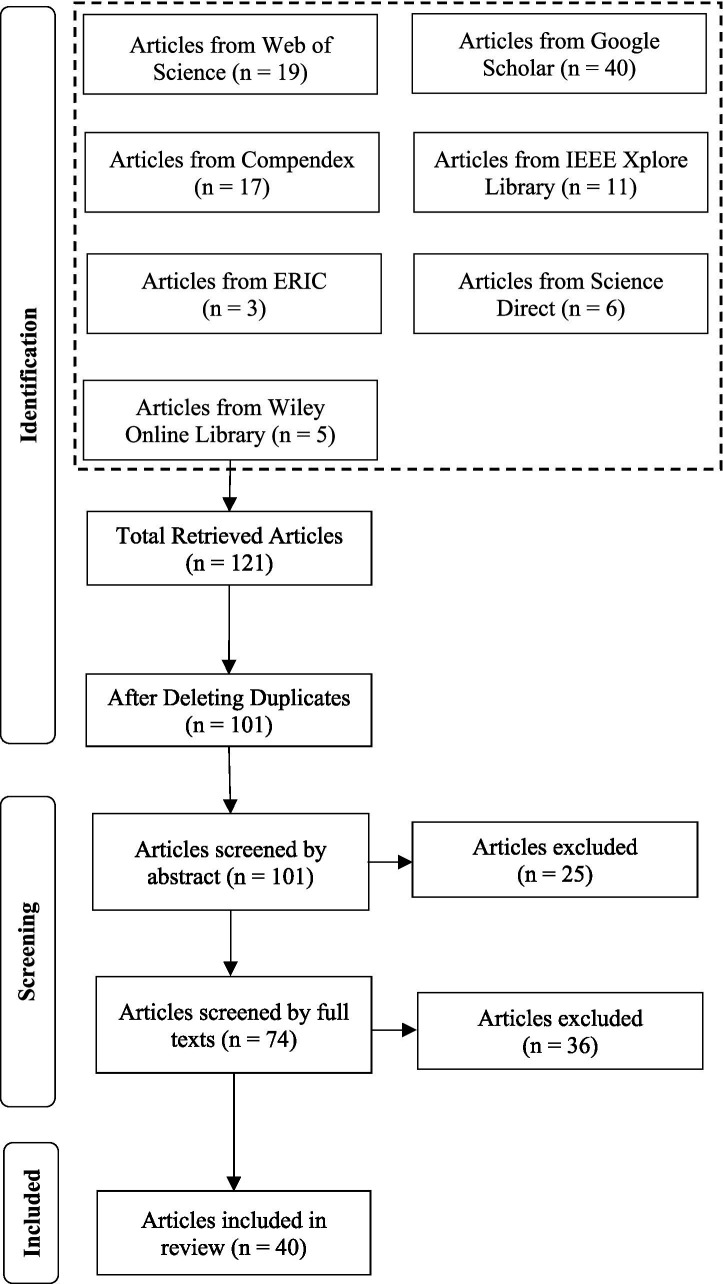
Systematic literature review article selection process using PRISMA (Page et al., 2021).

The complete article selection process for the SLR is presented in Figure 1. Articles meeting any of the following five exclusion criteria (EC) were removed because they did not meet the purpose of the study (EC1) Articles published before 2014 will be excluded; (EC2) Articles not published in English will be excluded; (EC3) Articles that do not focus on chatbot design will be excluded; (EC4) Articles that do not focus on chatbot design using ChatGPT will be excluded; (EC5) Articles that are work-in-progress or short papers will be excluded.

As shown in Figure 1, 121 articles were initially retrieved using search phrases and seven databases. To conclude the identification phase, we removed duplications among articles pulled from various databases and then completed the initial screening of abstracts and, later, full articles to eliminate articles that were easily identifiable as meeting one or more of the exclusion criteria. The remaining articles were evaluated independently for inclusion. The remaining articles were reevaluated against the exclusion criteria. Forty articles were ultimately included in the final synthesis phase of the review.

### Data analysis

With the final 40 articles screened, the authors followed a six-step process to analyze and generate meaning from the publications. The articles were independently read and summarized. The following information that was documented from each article included, title, year published, country of the first author, research question, methodology, chatbot design methods, findings, implications, limitations, future work, important notes, motivation for research, algorithms used, data collected, prompts used, and generative AI used. Lastly, after analyzing and comparing the data recorded from all articles, codes were generated to describe common patterns across the 40 articles. Eight codes were generated: user experience, engagement, relationship, interaction, hybrid solution, prompt fickleness, prompt, and complex prompts.

### Strengths and limitations

This SLR provides a comprehensive view over framework for designing intelligent chatbots using ChatGPT. An in-depth analysis is used to shed light on the potential limitations of chatbot design using ChatGPT as well as the endless opportunities the system has to offer. Each theme generated as part of this study is accompanied by implications for both practice and research to provide developers and researchers who are interested in designing chatbots with ChatGPT specific and actionable guidance. The findings of this study greatly expand the current understanding of designing chatbots with ChatGPT as they help categorize the strengths of existing research and identify opportunities for future research. While limited systematic literature reviews have focused specifically on ChatGPT-based chatbot design, recent surveys (e.g., Casheekar et al., 2024; Cerny, 2023) have expanded on various categories of LLM-powered conversational agents. This review differs in area and method by focusing only on ChatGPT implementations and combining both technical and user-experience aspects within a unified framework.

Regarding limitations, the final set of articles was selected based on exclusion criteria that did not account for the quality or uniqueness of the information they contained. While we used seven respected databases (Google Scholar, IEEE Xplore, ERIC, ScienceDirect, Compendex, Wiley Online Library, and Web of Science), the initial search relied primarily on the phrase “Chatbot design + ChatGPT.” This may have excluded relevant studies that used different terminology, such as “conversational agents,” “dialogue systems,” “LLM-based virtual assistants,” or “generative AI-driven bots.” Additionally, during retrieval, many potentially relevant articles appeared that focused on chatbot design using large language models (LLMs) but not specifically ChatGPT, as a result these were excluded under the current criteria. No formal bias or quality appraisal tools (e.g., AMSTAR, CASP) were applied in this review. While the exclusion criteria (EC1–EC5) ensured subject relevance and basic methodological reliability, future reviews could incorporate formal quality assessments to further refine validity. Future systematic reviews could broaden the scope to include LLM-powered chatbots in general, which may provide a more thorough image of design approaches and outcomes. Future reviews may also benefit from an expanded search strategy that includes broader terms to reduce the possibility of missing relevant literature. We also excluded books and technical reports, which may have further limited the scope of the review.

### Findings

We first present a descriptive summary of trends in research and practice chatbot design using ChatGPT. Then, we provide descriptions, exemplary studies, and research and practice implications for the three thematic questions identified from synthesizing the final 40 articles.

#### Country affiliation of first author

Table 2 shows the articles that were selected for this review containing the first authors from 23 different countries. The majority of first authors came from the United States of America (20%). Countries such as Turkey and China developed a decent portion each with 10%. South Korea follows with 7.5%. Saudi Arabia and Italy each contained 5% as the remaining countries each have a value of 2.5% (The United Kingdom, Sweden, Egypt, Indonesia, India, Czech Republic, Greece, Lebanon, Australia, Japan, Peru, Netherlands, Oman, Thailand, Croatia, Finland, and Israel). Acknowledging the selective number of articles used we can still perceive that chatbot design with ChatGPT is more frequent between the United States with a well standing portion lying in countries like Turkey, China, Saudi Arabia, South Korea, and Italy. Although there is still a distinct variety of countries presented in Table 2, implicating the broad outreach of chatbot design with ChatGPT. This analysis is based solely on first author affiliations and does not capture the full collaborative nature of the research. Co-author affiliations, institutional partnerships, and funding sources were not analyzed, which may underrepresent the international collaborative scope of this research domain.

**Table 2:** Country affiliation of first author of the sampled articles.

#	Country	*N*	%
1	USA	8	20
2	Turkey	4	10
3	China	4	10
4	South Korea	3	7.5
5	Italy	2	5
6	Saudi Arabia	2	5
7	Sweden	1	2.5
8	Egypt	1	2.5
9	Indonesia	1	2.5
10	India	1	2.5
11	Czech Republic	1	2.5
12	Greece	1	2.5
13	United Kingdom	1	2.5
14	Lebanon	1	2.5
15	Australia	1	2.5
16	Japan	1	2.5
17	Peru	1	2.5
18	Netherlands	1	2.5
19	Oman	1	2.5
20	Thailand	1	2.5
21	Croatia	1	2.5
22	Finland	1	2.5
23	Israel	1	2.5

#### Motivation for research

Understanding the intended purpose and application domain of chatbots is essential for evaluating their design effectiveness and user satisfaction. The reviewed articles demonstrate that chatbots are increasingly employed across diverse domains, each requiring specialized conversational approaches and domain-specific ethical considerations. Based on Table 3 chatbots that had the intended design to handle emotional and health queries (Florindi et al., 2024; Alessa and Al-Khalifa, 2023; Lee et al., 2024; Wang H. et al., 2024, Li et al., 2024; Triantafyllopoulos et al., 2024; Kim, 2023; Mendoza-Pinto, 2023; Shapiro and Lyakhovitsky, 2024) consisted of 22.5% of the accepted articles. These articles relate to a chatbot that produces responses revolving around emotional or health assistance. Chatbots that were designated to be used as educational assistants (Koyuturk et al., 2023; Islam et al., 2024; Khadija et al., 2023; Yang et al., 2024; Tayan et al., 2024; Ambikairajah et al., 2024; Cerny, 2023; Ciampa et al., 2024; Gill et al., 2024; Hallal et al., 2023; Pack and Maloney, 2023; Tripaldelli et al., 2024; Jeon et al., 2023; Kipp et al., 2024; Rajala et al., 2023) composed 37.5%. Articles that utilize the chatbot to address capabilities and limitations chatbot response and design compiled 22.5% (Thapa and Patel, 2024; Zamfirescu-Pereira et al., 2023; Ekvall and Winnberg, 2023; Wei et al., 2024; Bettayeb et al., 2024; Casheekar et al., 2024; Heidari et al., 2024; Madunić and Sovulj, 2024; Salah et al., 2024).

**Table 3:** Different usages of chatbot in the sampled articles.

Designated usage of chatbot	Description	*N*	%
Emotional/Health assistance	Chatbots that intended use is for emotional assistance or care for the user	9	22.5
Educational assistance	Chatbots that intended use is to educate or aid toward education for the user	15	37.5
Business	Chatbots that intended use is for business applications ex. customer service	7	17.56
Addressing capabilities and limitations	Chatbots that intended use is for understanding limitations and capabilities within chatbot response and design	9	22.5

These chatbots can be used to determine the potential strengths and weaknesses between chatbot responses and design for future research purposes. Finally, chatbots that were designated to be utilized for business queries (Gallo et al., 2023; Islam et al., 2024; Kim et al., 2024; Limna et al., 2023; Martinez-Araneda et al., 2023; Okulu and Muslu, 2024; Young and Shishido, 2023) consisted of 17.5%. These chatbots were utilized to handle encounters in a work environment area such as customer service. After reviewing the following table, it is noticeable that chatbots designed to handle emotional/health queries are quite desired. It should also be addressed that (Islam et al., 2024) was included in two categories (business, educational assistance) as it focuses on improving customer service, managing inquiries, and increasing efficiency, all of which are essential in business settings. The chatbot’s role in helping users navigate services and components such as theaters and halls within an educational institution ties it directly to educational support.

#### Algorithms, technologies, methods, and tools used

Across each of the selected articles’ algorithms were explicitly mentioned or at least briefly explained. Algorithms, technologies, methods, and tools play a fundamental role in chatbot design as they provide logic and processes that allow the chatbot to understand and respond to user inputs effectively. Without these resources, chatbots would be unable to process language, understand meaning, and create accurate responses. Table 4 provides an understandable view of diverse algorithms, technologies, methods, and tools used. A trend that can be indicated is that ChatGPT carries a central role among each of the articles especially toward creating responses and processing inputs. ChatGPT’s versatility has the capability to cover areas like prompt engineering, zero-shot learning, and AI chaining, showcasing its ability to handle diverse tasks such as emotional detection, generating responses, and text classification. This highlights the growing attachment to advanced pre-trained language models (LLMs) for developing intelligent chatbot systems.

**Table 4:** Algorithms, technologies, methods, and tools used in the sampled articles.

#	Algorithms, technologies, methods, and tools used	Reference article
1	Prompt engineering, rule based UX prototyping, and iterative prototyping of prompts, ChatGPT	Zamfirescu-Pereira et al. (2023)
2	ChatGPT used to understand and generate responses, prompt tuning, cipher query language	Thapa and Patel (2024)
3	NLP, Rasa, prompt engineering, ChatGPT	Gallo et al. (2023)
4	EmoRoBERTa for emotional detection, ChatGPT for responses, LangChain, BertScore, UNIEVAL, Google Search API	Florindi et al. (2024)
5	ChatGPT, AI chaining, prompt engineering	Ekvall and Winnberg (2023)
6	ChatGPT, Zero-shot learning, prompt engineering, iterative design framework	Koyuturk et al. (2023)
7	ChatGPT, ASR, TTS, prompt engineering	Alessa and Al-Khalifa (2023)
8	ChatGPT, FAISS, NLP techniques	Lee et al. (2024)
9	ChatGPT, CNN for text classification, LSTM	Wang H. et al. (2024)
10	Natural language understanding (NLU), natural language generation (NLG), ChatGPT	Islam et al. (2024)
11	LLMs, Vector storage and similarity scoring, LangChain, BLEU score, ChatGPT	Khadija et al. (2023)
12	NLP (natural learning processing) techniques, AI, ML (machine learning)	Cerny (2023)
13	ChatGPT, natural language queries, ML, deep learning, transformer-based models	Kipp et al. (2024)
14	GPT 3.5, 4, NLP, LLMs, Elicit, Iris.ai, automated speech recognition (ASR) machine translation, dynamic written corrective feedback	Pack and Maloney (2023)
15	GPT-3, GPT-4, Self attention and positional encoding, pre-training, fine tuning, retrieval based and generative AI techniques, reinforcement learning from human feedback (RLHF)	Heidari et al. (2024)
16	LLMs, RLHF, neural networks, NLP, data collection and statistical analysis methods	Triantafyllopoulos et al. (2024)
17	LLMs, deep learning, NLP, machine learning, bias mitigation and ethical AI considerations	Ciampa et al. (2024)
18	GPT-4. NLP, fine tuning	Wei et al. (2024)
19	NLP, ChatGPT, machine learning	Hallal et al. (2023)
20	ChatGPT, prompt engineering, integrated circuit design, machine learning	Yanık et al. (2023)
21	ChatGPT, Student feedback	Ambikairajah et al. (2024)
22	Teaching machine learning based on BiLSTM, ChatGPT, Deep Neural Network, SMOTE, WGAN, KNN	Zhou (2023)
23	ChatGPT, prompt engineering	Tripaldelli et al. (2024)
24	ChatGPT, prompt engineering, NLP, LLMs	Young and Shishido (2023)
25	LLMs, ChatGPT, NLP	Mendoza-Pinto (2023)
26	RLHF, ChatGPT, LLMs, fine-tuning	Tuan et al. (2024)
27	ChatGPT, NLP, LLMs, prompt engineering	Okulu and Muslu (2024)
28	Machine learning, ChatGPT, Fine-tuning	Yang et al. (2024)
29	Structural equation modeling (SEM), ChatGPT, fine-tuning	Kim et al. (2024)
30	LLMs, prompt engineering, fine-tuning, ChatGPT	Jeon et al. (2023)
31	ChatGPT, LLMs, SEM, SmartPLS	Salah et al. (2024)
32	NLP, Reinforcement Learning, ChatGPT, fine-tuning	Kim (2023)
33	ChatGPT, UI/UX design analysis, LLMs, fine-tuning	Casheekar et al. (2024)
34	Prompt engineering, LLMs, ChatGPT	Gill et al. (2024)
35	ChatGPT, fine-tuning, deep learning, machine learning	Limna et al. (2023)
36	ChatGPT, NLP, WSN, description	Tayan et al. (2024)
37	ChatGPT, LLMs, NLP, fine-tuning, prompt engineering	Madunić and Sovulj (2024)
38	NLP, machine learning, ChatGPT, learning analytic systems, fine-tuning	Rajala et al. (2023)
39	ChatGPT, LLMs, dialogflow, BERT, prompt engineering	Topsakal and Topsakal (2022)
40	GPT, prompt engineering, fine-tuning, NLP	Shapiro and Lyakhovitsky (2024)

A second interesting finding is the integration of complementary NLP frameworks and tools. For example, EmoRoBERTa (Florindi et al., 2024) is utilized for emotional detection, indicating a focus on building emotionally aware chatbots. Tools like LangChain and Bert Score appear with ChatGPT to enhance the chatbot’s ability to generate, evaluate, and refine responses. The mix of these tools shows an important trend where systems depend on tool chaining and vector-based methods for improved understanding and performance. Also, text classification techniques, like CNN (Convolutional Neural Networks) and LSTM (Long Short-Term Memory), featured in Wang H. et al. (2024), show that deep learning architectures are still useful in text processing tasks. Meanwhile, techniques like FAISS (Lee et al., 2024) and vector storage (Khadija et al., 2023) show an increasing focus on similarity-based scoring and retrieval methods. These are essential for improving chatbot response relevance and search-based conversational models.

Finally, natural language understanding (NLU) and natural language generation (NLG) are explicitly mentioned in Islam et al. (2024), showing the importance of balancing language comprehension with high-quality response generation in intelligent chatbots. This balanced design allows the chatbots to understand user intent while also creating relevant and smooth replies. In summary, Table 4 showcases three main trends: the influence and power of ChatGPT as a base model, integration of specialized tools and NLP techniques to expand capabilities, and the use of deep learning and vector-based methods to improve classification and retrieval tasks. The prevalence of ChatGPT across studies is expected given the search criteria used. Therefore, results are reported objectively without interpreting frequency as influence. This knowledge is critical for researchers intending to design strong and intelligent chatbot systems that utilize modern AI frameworks and tools.

#### Thematic question analysis

This portion addresses three recurring thematic questions that were brought up in the articles. The three thematic questions that will be introduced are “How do user experience and engagement influence chatbot’s performance? “How do hybrid design models improve chatbot performance?,” and “What are the limitations of using ChatGPT, and how does prompt engineering affect responses?.” As we dive into these concepts, we will uncover how the articles cluster together, provide detailed examples revolving how the articles discuss the given topic, and finalize with a summary of the implications of the theme for future research and practical applications. The example articles provided were selected based on how the thematic question was focused on the article. Table 5 describes each thematic question, the codes linked to the specified topic and the number of articles that were designated to the related question.

**Table 5:** Thematic questions, descriptions, and associated codes and sampled articles.

Thematic questions	Definition	Codes	*N*
How do user experience and engagement influence chatbot’s performance?	Topics that describe users’ experience with the tested chatbot, including engagement, interactions, and using the chatbot.	Users’ experience engagement relation interaction	17
How do hybrid design models improve chatbot performance?	Topics that discuss a hybrid design framework toward the creation of the chatbot.	Hybrid design dual structure	9
What are the limitations of using ChatGPT, and how does prompt engineering affect responses?	Topics that discuss the limitations of that chatbot related to prompt structure. Specifically handling complex prompts and prompt sensitivity.	Prompt fickleness Prompt sensitivity Complex prompts Prompt Limitations	14

### Question 1: how do user experience and engagement influence chatbot’s performance?

When designing a chatbot, it is essential for users to feel directly addressed, as this helps them clearly recognize and engage with the chatbot. User experience (UX) refers to the general experience a person has when interacting with a product or in this case the chatbot. A well-established UX helps ensure user satisfaction, efficient interactions, and enjoyable/valuable exposure. Seventeen out of 40 articles discussed the implications of UX and user engagement within chatbot design. These 17 articles answer the question ‘*How do user experience andengagement influence chatbots’ performance*? Data that was collected to understand the effectiveness of the chatbot’s UX and engagement consisted of interaction data, user surveys, educational assessment, experimenter analysis, workshop observations, autobiographical design logs, user feedback, and artifacts. These 17 articles align with UX and dive deeper into how this influences the overall performance of the chatbot. These articles unfold the importance of UX throughout chatbot design (Zamfirescu-Pereira et al., 2023; Islam et al., 2024; Ekvall and Winnberg, 2023; Koyuturk et al., 2023; Thapa and Patel, 2024; Alessa and Al-Khalifa, 2023; Florindi et al., 2024; Martinez-Araneda et al., 2023; Wang X. et al., 2024; Khadija et al., 2023; Kim et al., 2024; Salah et al., 2024; Wei et al., 2024; Casheekar et al., 2024; Rajala et al., 2023; Tuan et al., 2024).

#### Exemplar studies

Studies illustrating UX and user engagement in chatbot performance were further analyzed, including Ekvall and Winnberg (2023). They developed the Prompt Board, a digital and collaborative tool resembling a mood board to help designers brainstorm and prototype chatbot functions using ChatGPT. Using an autobiographical design approach and a workshop with UX design students, the study explored how large language models can support UX design. Findings showed that UX and engagement significantly influence chatbot effectiveness. The Prompt Board’s visual interface helped idea generation, but users struggled with prototyping and needed more guidance. Participants felt that AI controlled most of the creative process, reducing their sense of ownership, although it also revealed new ways AI could support design. Some challenges included repetitive outputs and lack of real-world context. Overall, the study highlighted that strong UX design can enhance engagement and AI effectiveness, but addressing issues like creative control and AI limitations is essential. Designers often felt disconnected from the results, showcasing a tension between AI support and human creativity.

Koyuturk et al. (2023) explore how chatbots can be enhanced as educational tools, focusing on interactive, mixed-turn conversations about social media literacy tailored to user characteristics like age and culture. They developed a framework to evaluate ChatGPT’s ability to deliver engaging educational content while maintaining natural dialogue flow. The study highlights effective prompt strategies and challenges, such as the chatbot unexpectedly shifting roles (for example, from teacher to therapist). It found that well-crafted prompts significantly impact UX by keeping conversations relevant, engaging, and age-appropriate. Examples include a structured teaching loop prompt that combined explanation, assessment, and feedback, and another that personalized interaction based on age, tone, and cultural context. These prompts enabled adaptive, engaging learning experiences. Overall, the study shows that thoughtful prompt design can enhance chatbot-based education by supporting structured and flexible interactions that align with users’ learning needs.

#### Research implications

Future research implications based on this theme could be directed toward the importance of prompt engineering to shape chatbots or AI behaviors that align with specific UX goals. For example, in educational contexts, prompts can guide the chatbot to adopt teaching styles that suit various learning needs/preferences, ages, and cultural backgrounds. This opens avenues for research into how prompts can be designed or optimized to adapt across different user demographics and interaction types in educational or support-focused chatbots. Both articles reveal challenges with maintaining conversational continuity. Educational chatbots, for instance, may lose the intended teaching narrative over longer dialogues or switch roles (switching between assistant roles ex. Educational informative and emotional support).

The research findings discussed in Thapa and Patel (2024) have implications for the advancement and utilization of chatbots using Large Language Models (LLMs) such as ChatGPT. The study highlights the benefits of using domain-specific knowledge in LLM-based chatbots to improve accuracy and reduce errors, known as “hallucinations,” across various fields. The AI integrated chatbot used in this study can provide more engaging and personalized experiences compared to traditional rule-based chatbots, potentially leading to increased user satisfaction and more effective interactions. Khadija et al. (2023) emphasizes efficient document processing, like splitting and vector storage, to improve chatbot response accuracy and speed, enhancing user experience. Platforms like Pinecone offer opportunities for enhancing data management in large scale chatbots. It also highlights the importance of natural language understanding for effective and engaging chatbots. Using LLMs like GPT-3.5 Turbo allows more natural and satisfying interactions. Dynamic responses based on specific document content personalize and improve user engagement. Research into methods of maintaining context within generative AI conversations could enhance UX by ensuring that the AI remains reliable and context-aware over extended interactions.

#### Practice implications

Practitioners should pay close attention to prompt design in both educational and ideation contexts. Prompt customization is essential to achieve the desired interaction style, such as keeping educational chatbots focused on specific teaching goals or ideation tools on relevant user needs. Designers and developers can create prompt libraries tailored to different UX goals, allowing easier selection and refinement of prompts that guide AI behavior in alignment with user expectations and interaction contexts. In design ideation tools, there is a need for UX workflows that empower users without undermining their control over outcomes. This balance is crucial in ideation and creative applications, where users may need more agency over AI-generated suggestions. UX professionals can integrate features that let users adjust or override AI suggestions, possibly through feedback loops where users refine AI outputs, thereby fostering a collaborative rather than an automated ideation process.

The studies show that AI systems that adapt to user demographics, such as age or cultural background, tend to increase relevance and engagement. For educational contexts, this could mean more personalized interactions that adapt to users’ specific learning preferences. Practitioners can build demographic or skill-based customization into chatbots, which tailor the AI’s tone, complexity, and content focus to each user. Regular feedback opportunities during interactions can also help in fine-tuning the AI’s responsiveness to individual preferences. Khadija et al. (2023) showcase how AI can enhance academic workflows by making information retrieval easier in educational settings. Its natural language processing enables intuitive interactions, providing personalized support for students and faculty. This can improve user experience and make learning and teaching more engaging. Thapa and Patel (2024) implies that AI-powered chatbots can understand natural language, making interactions more efficient and reducing frustration. They handle diverse queries, adapt to new information, and maintain consistent user experience as they develop. By using a knowledge graph, they minimize errors and build trust. Prompt tuning further enhances their performance over time, improving reliability and engagement.

### Question 2: How do hybrid design models improve chatbot performance?

The conventional approach to chatbot creation typically follows a single design model (Zamfirescu-Pereira et al., 2023). While straightforward, this method can lead to notable limitations due to its dependence on one specific framework. A single model is rarely capable of accommodating the full range of complexities in natural language processing, leading to constraints in flexibility, adaptability, and accuracy (Gallo et al., 2023). The potential limitations of a single design model include issues with handling ambiguous queries, understanding context, and scaling across diverse user needs (Zamfirescu-Pereira et al., 2023). Hybrid design integration allows for a more well-rounded chatbot design. Hybrid design models typically combine multiple approaches to enhance the chatbot’s efficiency. This is due to the usage of a multi structure that combines the strengths of two different models to enhance chatbot design. This design structure also has the potential to cancel out any limitations from a single model. This proposal raises the question “How do hybrid design models improve chatbot performance?.” To answer this, a comprehensive analysis of nine articles focused on hybrid chatbot design models was conducted. These sources provided detailed insights into the ways in which hybrid models address and reduce limitations seen in single model designs. Articles emerged from key themes on the benefits of hybrid design, particularly in how these models overcome common issues such as context retention, scalability, and interaction complexity. These articles go in depth on how the strengths of a hybrid design model can reduce the limitations of chatbot performance (Zamfirescu-Pereira et al., 2023; Gallo et al., 2023; Florindi et al., 2024; Wang H. et al., 2024).

#### Exemplar studies

Articles highlighting the impact of hybrid design models were analyzed to clarify the thematic question. Gallo et al. (2023) explored this by combining ChatGPT and Rasa in developing a smart chatbot for managing trigger-action rules in smart homes. The hybrid approach takes advantage of ChatGPT’s strength in handling complex language tasks like multi-step commands and conversational breakdowns, while Rasa manages intent recognition, entity extraction, and structured dialogue. This combination allows for better context retention, smoother interactions, and more relevant responses. A similar sentiment-analysis chatbot using LangChain and DialogFlow maintained memory for emotionally aligned, context-aware replies. The hybrid model balances adaptability (ChatGPT) with structured accuracy (Rasa), enhancing scalability and handling complex commands. Especially, when Rasa failed to interpret a command, ChatGPT could rephrase it to provide a coherent response making interactions more intuitive. The chatbot also adapted responses based on users’ specific smart home setups, improving relevance and user experience.

Florindi et al. (2024) present a hybrid approach to building an emotionally aware chatbot by integrating ChatGPT with sentiment analysis and information retrieval tools. The system combines text generation with models like EmoRoBERTa for emotion detection and frameworks such as LangChain and DialogFlow to deliver empathetic, context-aware responses. Their chatbot, “F-One,” was tested in the Formula 1 domain, providing accurate and emotionally aligned answers about rules and events. Evaluation tools like BERTScore and UNIEVAL confirmed its clarity and contextual accuracy. ChatGPT offered flexible, emotion-sensitive replies, while the retrieval model ensured domain-specific accuracy. The study demonstrates the potential of emotionally aware chatbots for customer service, business, and emotional support. While the system performed well, challenges remained in maintaining consistent performance across conversations. The hybrid design effectively addressed common issues in single-model chatbots such as limited emotional understanding and context retention, highlighting the benefits of combining generative and retrieval-based approaches.

#### Research implications

Research implications highlight the value of hybrid design in chatbot development. By combining multiple models, hybrid chatbots outperform single-model systems in context retention, accuracy, and emotional intelligence. These systems assign tasks across specialized models, for example ChatGPT for natural language generation and Rasa for rule-based intent handling, enabling more effective domain-specific adaptation, such as in smart homes or customer support. Hybrid models also enhance emotional engagement. Integrating tools like EmoRoBERTa for emotion detection with ChatGPT allows for empathetic, adaptive responses beneficial in areas like mental health and customer service. Zamfirescu-Pereira et al. (2023) emphasizes the need for further research on prompt-based and hybrid systems, specifically in optimizing the mix of prompts, machine learning, and rule-based logic. Tan et al. (2024) supports this, showing that combining tools like ChatGPT, CNN, and LSTM improves chatbot performance. Their findings also reveal that 70% of users prefer giving feedback by chatbots, pointing to the importance of studying user preferences and interaction styles to inform better chatbot design.

Researchers also need to develop ways to measure how reliable, flexible, and user-friendly hybrid chatbots are compared to single-model ones. Finally, understanding how prompts work with other system parts can help improve chatbot performance. By studying these areas, we can overcome the limits of prompt-based systems while using the power and flexibility of LLMs effectively. These research findings suggest that hybrid models can significantly expand the chatbot’s capabilities, making them more accurate, contextually aware, and emotionally responsive compared to single model designs. As chatbots become more widely used across varied applications, optimizing these hybrid models for scalability, speed, and adaptability will be essential.

#### Practice implications

The practical implications of hybrid chatbot designs, as discussed in recent studies, demonstrate how combining models enhances chatbot performance in real-world applications. By integrating tools like ChatGPT for natural language processing and Rasa for intent recognition, hybrid chatbots can manage both structured tasks and open-ended queries more accurately (Gallo et al., 2023). This hybrid capability reduces misinterpretation, especially in customer service, where clear communication is critical. Hybrid systems also adapt well to specialized domains, such as smart homes or regulatory contexts, by pairing flexible language models with rule-based logic. For example, in a smart home setting, ChatGPT can handle broad requests while Rasa carries out specific commands, improving reliability and reducing errors (Gallo et al., 2023). Emotionally intelligent hybrid chatbots further improve user satisfaction in high-empathy areas like mental health or customer support. Combining EmoRoBERTa for emotion detection with ChatGPT for adaptive responses creates more human-like, emotionally aware interactions (Florindi et al., 2024), supporting trust and user engagement. Zamfirescu-Pereira et al. (2023) argue that prompt-based designs alone often fall short and suggest combining them with structured dialogue or ML models to improve reliability. Hybrid methods allow for flexible improvements without needing to redesign entire systems. Tan et al. (2024) emphasize the value of hybrid chatbots in healthcare, noting benefits like real-time support, better handling of sensitive issues, and improved accessibility across diverse populations. Integrating with existing healthcare platforms enhances adoption and efficiency. All together, these findings show that hybrid chatbot designs overcome the limitations of single-model systems while enabling more accurate, adaptive, and emotionally intelligent user experiences across various domains.

### Question 3: What are the limitations of using ChatGPT, and how does prompt engineering affect responses?

When designing a chatbot it is important to address limitations and how different prompts affect the responses of the chatbot. Addressing these concerns can lead to a better understanding of the performance of the chatbot while continuing to find ways to improve the execution. Prompts directly shape the chatbot’s responses. Different prompts can guide the chatbot toward specific types of answers, affecting the clarity, relevance, and depth of the responses generated. A well-developed prompt can help the chatbot focus on the user’s intent, improve response accuracy, and handle ambiguous questions more effectively. In contrast, poorly designed prompts may result in generic, irrelevant, or even incorrect responses. In some cases, experimenting with prompt phrasing can help overcome the limitations of the underlying model, such as by encouraging the chatbot to provide explanations or break down complex instructions into simpler steps. As chatbots become more common in customer support, education, and personal assistance, refining prompts to match the intended user experience is essential for delivering reliable, contextually appropriate interactions (Zamfirescu-Pereira et al., 2023). This develops the question of “What are the limitations of using ChatGPT, and how does prompt engineering affect responses?.” To understand this query articles used had to fit within the criteria of diving into the effects of prompt sensitivity and fickleness. These articles go into depth on the importance of prompt design and potential limitations (Zamfirescu-Pereira et al., 2023; Koyuturk et al., 2023; Thapa and Patel, 2024; Gallo et al., 2023; Ekvall and Winnberg, 2023; Alessa and Al-Khalifa, 2023; Lee et al., 2024).

#### Exemplar studies

Alessa and Al-Khalifa (2023) looks at using a ChatGPT-based system to help reduce loneliness and isolation in elderly people. Their system combines speech recognition, ChatGPT, and text-to-speech technology to make it an easy tool for older adults. After testing, they found that prompts with a medium level of detail, based on personal information like the user’s background and interests, provided a good balance between personalization and efficiency. Tests with simulated elderly users showed that the system could give relevant and engaging responses, which experts rated based on qualities like interest and smoothness. However, the study notes some challenges, such as risks of bias and misinformation, privacy concerns, limited emotional understanding by the chatbot, and the chance that elderly users might rely too much on it for social interaction. They also found that prompt detail affects conversation quality. Detailed prompts make conversations more personalized but use more tokens, while medium-detailed prompts are more efficient. Including conversation history also helps keep conversations on track. Although the system shows stability, the study highlights the need to address ethical and design challenges before using it widely.

The article Zamfirescu-Pereira et al. (2023) discusses the challenges and limitations of designing a chatbot using GPT-3 prompts. The study explored how ChatGPT could be used to create interactive chatbots for teaching social media literacy. The researchers focused on designing prompts that could adapt to users’ needs and handle multi-turn conversations. They found that the chatbots were able to adjust educational content based on characteristics like age and culture, making learning more personalized. However, challenges included inconsistent conversational styles, rigid responses, and unexpected behavior shifts, such as the chatbot acting like a therapist or dictionary. Structured prompts helped chatbots use humor and connect better with users from different cultures. However, ChatGPT’s memory limits made it hard to keep track of long conversations. The study suggested integrating these chatbots with larger learning systems to improve their educational abilities and fix these issues. This study finds that while prompt-based chatbot design can meet about 80% of user experience (UX) goals, the remaining 20% often require extensive adjustments and are difficult to achieve reliably. Prominent limitations include the chatbot’s inability to consistently say “I do not know” when it should, leading to misleading or incorrect answers, and the fickleness of prompts, where effective prompts can become ineffective when combined with others. This limits the chatbot’s reliability, particularly in complex or high stakes scenarios. Different prompts significantly affect responses by controlling the chatbot’s behavior, tone, and conversational style. However, designing prompts requires careful iteration and balancing specificity with adaptability. The study suggests that highly prescriptive prompts help control chatbot responses but risk making interactions overly scripted, which can reduce the spontaneity and fluidity expected in a conversational interface.

#### Research implications

The research implications discussed in the articles (Zamfirescu-Pereira et al., 2023; Koyuturk et al., 2023; Thapa and Patel, 2024; Gallo et al., 2023; Ekvall and Winnberg, 2023; Alessa and Al-Khalifa, 2023; Lee et al., 2024) reveal that while prompting is a powerful tool for guiding chatbot responses, it has limitations that impact its usability and control in UX design. Prompts influence chatbots significantly by determining conversational tone, pacing, and response style. However, the fickleness of prompts means that minor adjustments or combinations with other instructions can lead to unpredictable results, complicating the design process. For example, prompts meant to elicit a specific response might work well in isolation but fail when integrated into a larger conversational flow. This unpredictability requires designers to prototype and test every prompt iteration extensively. The findings highlight that achieving complex UX goals with prompt only approaches are challenging, particularly in high-stakes domains like medicine or law, where reliability is crucial. Researchers suggest that prompt-based designs may be better suited for lower risk applications until further advances are made. Additionally, the challenge of reliably making the bot say “I do not know” illustrates the limitation of prompt-based control, as even extensive prompt engineering could not yield consistently accurate responses for uncertain answers. In summary, the research implicates for a deeper exploration into tools and methodologies that could help visualize and manage the interactions between different prompts, potentially through iterative design tools that aid in balancing spontaneity with reliability. This could pave the way for more significant and controllable conversational components in future applications.

#### Practice implications

The practical implications from the articles (Zamfirescu-Pereira et al., 2023; Koyuturk et al., 2023; Thapa and Patel, 2024; Gallo et al., 2023; Ekvall and Winnberg, 2023; Alessa and Al-Khalifa, 2023; Lee et al., 2024) emphasize that designing effective chatbot prompts requires careful and iterative work due to the fickleness of prompts. Even small changes in prompt phrasing or ordering can greatly alter chatbot responses, affecting consistency and reliability. This is particularly challenging because adding new prompts or combining them with existing ones may disrupt previous behaviors, leading designers to address multiple issues at the same time, often in unpredictable ways. This unpredictability means that prompt-based chatbot design is resource intensive, requiring constant testing and fine tuning. To avoid errors or unintended responses, designers are encouraged to use highly specific, prescriptive prompts. However, overly prescriptive prompts risk making the chatbot’s interactions feel rigid and scripted, which might sacrifice the conversational fluidity that users expect from AI. In practice, the articles suggest that prompt engineered chatbots may currently be best suited for low stakes where controlled scripted interactions are allowed. For more flexible or high stakes applications, designers may need to combine prompts with other techniques, like fine tuning or custom models, to manage random chatbot behaviors while ensuring reliability. These findings highlight the need for improved tools that help visualize and manage prompt interactions, enabling more streamlined and effective chatbot design processes.

### Negative findings and controversies

While many of the reviewed studies highlight the value of ChatGPT-based chatbots, several reported limitations, inconsistent results, and ongoing controversies. For example, Zamfirescu-Pereira et al. (2023) and Alessa and Al-Khalifa (2023) noted that heavy reliance on generative AI can lead to “hallucinations,” misinformation, or unreliable conversational style, which is mainly problematic in critical fields like healthcare and education. Studies by Lee et al. (2024) and Wang H. et al. (2024) emphasized ethical concerns such as bias in training data, absence of clarity in decision-making, and lacking safeguards for privacy and data security. In emotionally sensitive contexts, chatbots sometimes generated responses seen as inappropriate or lacking empathy, undermining user trust. Several articles also warned against the risk of users forming dependency on conversational AI for emotional support, which could have long-term psychological effects. Finally, researchers noticed that effective prompt strategies in one context could fail entirely when combined with other prompts, making the design process unstable and demanding significant resources. These findings show the importance of balancing innovation with care, especially in situations where reliability, safety, and ethical integrity are crucial.

### Framework for designing and implementing chatbots

**Figure 2:**
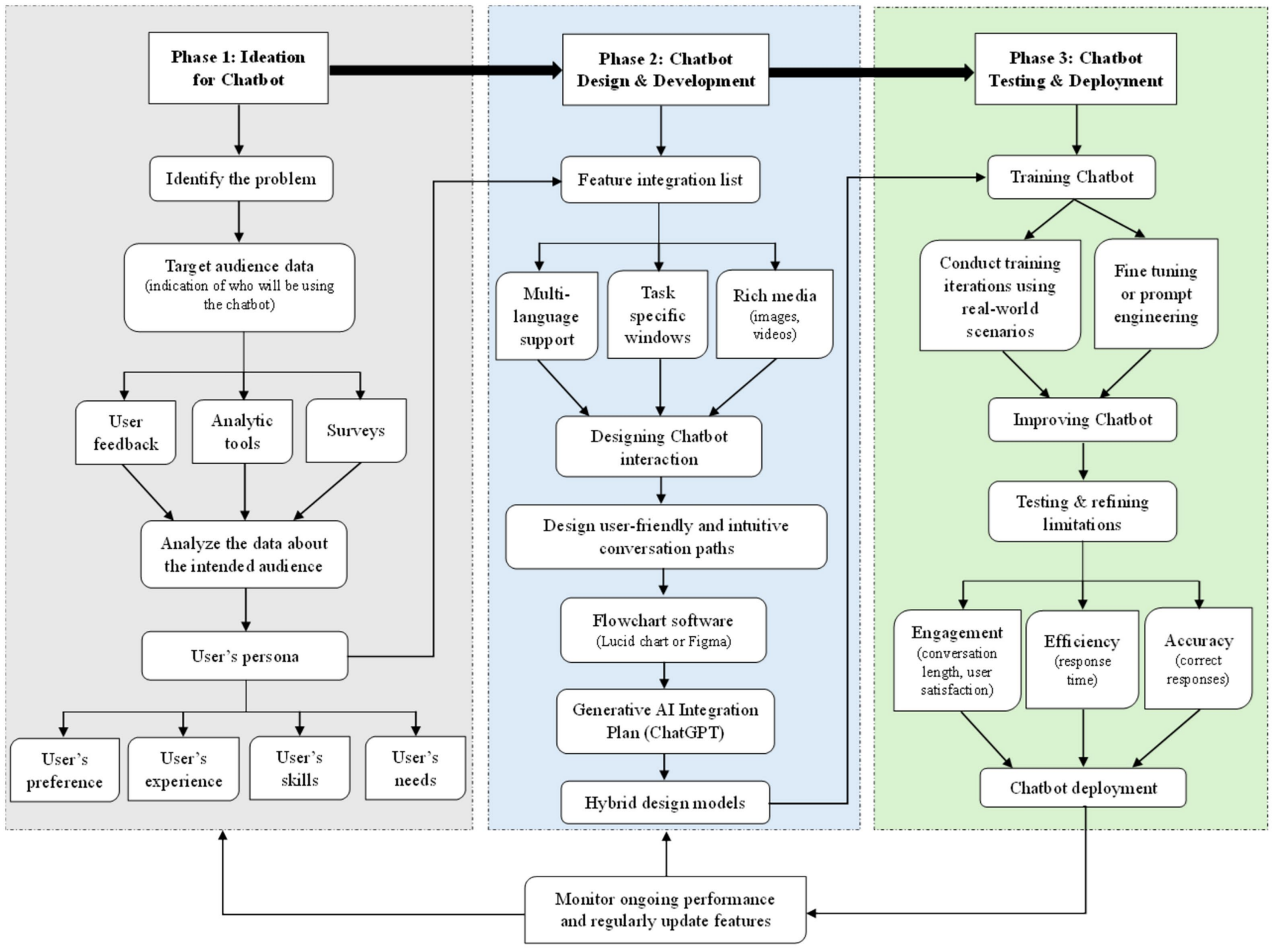
Framework for designing effective chatbots with ChatGPT.

The framework presented in Figure 2 is designed to assist beginners with chatbot design utilizing ChatGPT. It provides a comprehensive guide to understanding and implementing the various aspects of chatbot development, ensuring that even those with limited experience can navigate the process effectively. Chatbot design is a diverse initiative that involves careful consideration of various elements, ranging from conceptualization to implementation and ongoing refinement.

#### Phase 1: ideation for chatbot

The first phase in designing an effective chatbot begins with a structured ideation process, focusing on problem identification, audience analysis, and user persona development. Establishing a solid foundation in this phase ensures that the chatbot aligns with user needs, functions efficiently, and enhances overall engagement.

##### Identifying the problem and defining the chatbot’s purpose

Before designing a chatbot, it is essential to clearly define the problem it aims to solve. This step involves assessing pain points, inefficiencies, or repetitive tasks within a specific domain where a chatbot could provide value. The chatbot’s purpose must be well-articulated, as it serves as the guiding principle for the entire development process (Alessa and Al-Khalifa, 2023; Casheekar et al., 2024). Questions to consider at this stage include: What specific challenges does the chatbot aim to address? Will the chatbot provide information, automate tasks, or enhance user interactions? How will it contribute to efficiency, engagement, or accessibility in its intended domain? A well-defined purpose ensures that the chatbot is goal-oriented and effectively meets user expectations. Without a clear objective, the chatbot may lack direction and fail to deliver meaningful interactions (Gallo et al., 2023; Heidari et al., 2024).

##### Identifying and analyzing the target audience

Once the problem and purpose are established, the next critical step is determining the target audience. A chatbot designed for customer service in e-commerce will have different requirements than one built for educational support in universities. Understanding who will use the chatbot allows developers to customize its functionalities, interaction style, and content delivery. Key aspects to consider when defining the target audience include: (a) Demographics—Age, gender, language preference, location, and cultural background. (b) Industry or Sector—Will the chatbot serve a corporate, healthcare, educational, retail, or entertainment domain? (c) Skill Level and Technological Proficiency—Will users be tech-savvy professionals, students, or individuals with limited digital experience? (d) User Needs and Expectations—What are the common challenges or queries users face, and how can the chatbot address them effectively? A well-defined target audience helps in shaping the chatbot’s tone, language, accessibility features, and interaction pathways, ensuring that it delivers a personalized and intuitive user experience (Gill et al., 2024; Islam et al., 2024).

##### Collecting and analyzing audience data

To gain data-driven insights into the target audience, various data collection methods can be employed: (a) User Feedback and Surveys—Directly engaging with potential users through questionnaires or interviews to gather insights about their expectations. (b) Analytical Tools—Using web analytics, heatmaps, and engagement reports to study user behavior patterns. (c) Market Research Reports—Examining industry-specific trends and best practices for chatbot applications. (d) Competitor Analysis—Reviewing similar chatbot solutions to identify gaps, strengths, and potential improvements. By leveraging data collection tools, chatbot developers can ensure that design decisions are based on real-world user behavior and not just assumptions (Khadija et al., 2023; Limna et al., 2023).

##### Creating user personas

After gathering and analyzing audience data, designers can create user personas, which are detailed representations of typical users. Each persona includes the following key elements: (a) Name and Background—A fictional yet realistic profile to represent a segment of the target audience. (b) Goals and Needs—What the user wants to achieve using the chatbot. (c) Pain Points and Challenges—Specific problems the user faces that the chatbot can address. (d) Preferred Communication Style—Whether the user prefers short, direct responses or detailed explanations. (e) Technological Proficiency—The level of comfort the user has with digital tools.

For example, if the chatbot is designed for a university helpdesk, a possible persona might be: *Name*: Alex, a second-year undergraduate student. *Needs*: Quick responses to administrative queries (e.g., course registration, deadlines). *Pain Points*: Long wait times for email responses and difficulty finding information. *Preferred Communication Style*: Short, direct answers with links to relevant resources. *Tech Proficiency*: Comfortable using online platforms but prefers mobile-friendly interfaces. Developing user personas helps chatbot designers anticipate user expectations, refine chatbot interactions, and create a more engaging experience (Cerny, 2023; Kim et al., 2024; Pack and Maloney, 2023).

##### Aligning chatbot design with user experience principles

A successful chatbot must be user-friendly and intuitive. The ideation phase must incorporate User Experience (UX) design principles to ensure smooth interactions. This involves: (a) Ensuring clarity in chatbot responses—Avoiding vague or misleading answers. (b) Providing structured conversation pathways—Designing intuitive flows so users can quickly navigate interactions. (c) Including accessibility considerations—Ensuring inclusivity for users with disabilities (e.g., voice-to-text options, screen reader compatibility). (d) Personalization elements—Allowing users to set preferences for tone, language, and interaction style. A chatbot that prioritizes UX ensures higher engagement, trust, and usability, making it more effective in serving its intended purpose (Adarsh, 2025; Ekvall and Winnberg, 2023; Kim, 2023).

The ideation phase lays the groundwork for chatbot development by identifying the problem, defining the target audience, analyzing user behavior, and creating personas. This structured approach ensures that the chatbot is designed with user needs in mind, enhancing its effectiveness and usability. The next phase will focus on chatbot design and development, where the insights gained from this phase will be used to build the chatbot’s features, interaction pathways, and AI integration (Ciampa et al., 2024; Rajala et al., 2023).

#### Phase 2: chatbot design and development

Once the problem and target audience have been defined, the next phase involves designing and developing the chatbot. This step focuses on structuring the chatbot’s functionality, interaction flow, and AI integration to ensure an efficient and user-friendly experience.

##### Listing essential chatbot features

To enhance clarity in problem-solving and ensure alignment with user expectations, it is crucial to define the Chatbot’s required features. This involves analyzing the chatbot’s objectives and user personas to create a detailed feature list. Features may include: (a) Multi-language support—if the chatbot will serve a diverse audience, integrating multiple language options can improve accessibility (Heidari et al., 2024). (b) Task-specific windows—for goal-oriented interactions (e.g., appointment scheduling, order tracking, or troubleshooting), structured windows can streamline navigation (Rajala et al., 2023). (c) Rich media integration—incorporating images, videos, buttons, and carousels can enhance interaction, making the chatbot more dynamic and engaging (Ambikairajah et al., 2024). (d) Personalization features—allowing users to customize preferences (such as conversation style or preferred topics) can improve engagement and user retention. By defining these key features early in development, designers ensure that the chatbot is functional, interactive, and well-equipped to assist users effectively (Gill et al., 2024; Kim et al., 2024).

##### Designing chatbot interactions

Chatbot interaction design determines how the chatbot will communicate with users, focusing on message flow, tone, and clarity. A well-structured interaction ensures that users can easily navigate conversations and accomplish their intended tasks without confusion (Ciampa et al., 2024). This process includes: (a) Conversation flow mapping—Structuring interactions using flowcharts helps visualize how users will engage with the chatbot at different stages. This process defines the chatbot’s responses to user inputs, redirections, and fallback mechanisms when the chatbot cannot understand a query (Alessa and Al-Khalifa, 2023). (b) Tone and language selection—Depending on the chatbot’s purpose, the tone can be formal (for professional applications like healthcare and finance) or casual (for entertainment and customer engagement). Maintaining clarity in responses is essential to prevent miscommunication (Pack and Maloney, 2023). (c) User-friendly conversation paths—Interactions should be intuitive and minimize user effort. Designing logical, goal-driven conversation flows ensures that users can efficiently receive assistance. Tools like Lucidchart or Figma can help visualize these conversation structures before implementation (Gallo et al., 2023).

##### Generative AI integration plan

The next step involves determining how ChatGPT or other generative AI tools will be integrated into the chatbot. This phase is critical in defining the chatbot’s intelligence, adaptability, and ability to generate meaningful responses. The integration plan involves: (a) Assigning ChatGPT’s role—Will ChatGPT handle all user interactions, or will it be combined with predefined rule-based responses? Clearly defining its role ensures seamless and consistent user experience (Casheekar et al., 2024). (b) Identifying potential limitations—ChatGPT’s ability to generate open-ended responses may sometimes lead to unpredictable or ambiguous answers. To mitigate this, developers may introduce hybrid design models, incorporating both generative AI and rule-based logic (Zamfirescu-Pereira et al., 2023; Patil et al., 2023). (c) Determining the need for additional AI tools—If ChatGPT has limitations in certain areas (such as image recognition or real-time data analysis), additional AI tools or APIs may be integrated to enhance functionality (Martinez-Araneda et al., 2023; Wang X. et al., 2024). (d) Aligning AI models with conversation flow—Ensuring that the chatbot follows the designed interaction pathways while leveraging AI-generated responses appropriately to avoid unnecessary complexity (Islam et al., 2024; Kim, 2023). This structured approach to AI integration guarantees that the chatbot remains responsive, context-aware, and capable of delivering accurate information (Rajala et al., 2023).

The chatbot design and development phase is essential for defining features, structuring interactions, and integrating AI capabilities. By carefully mapping out user conversations, selecting appropriate chatbot functionalities, and implementing AI strategically, this phase ensures that the chatbot is optimized for efficiency, usability, and engagement. The next stage will focus on testing and refining the chatbot to enhance its performance based on user feedback and real-world interactions (Limna et al., 2023; Thapa and Patel, 2024).

Any alternative text (alt text) provided alongside figures in this article has been generated by Frontiers with the support of artificial intelligence and reasonable efforts have been made to ensure accuracy, including review by the authors wherever possible. If you identify any issues, please contact us.

#### Phase 3: chatbot testing and development

Following the integration plan, the chatbot enters the critical phase of testing, fine-tuning, and deployment. This stage ensures that the chatbot functions as intended, provides accurate responses, and delivers a seamless user experience. By iterating through training, testing, and refinement, developers can optimize the chatbot for real-world use and long-term adaptability (Islam et al., 2024; Casheekar et al., 2024).

##### Training and fine-tuning the chatbot

Before real-world deployment, the Chatbot undergoes a structured training process to optimize its performance for specific tasks, industries, or user interactions. This step involves: (a) Prompt engineering—Carefully designing and refining prompts that guide the chatbot’s responses to ensure clarity, accuracy, and relevance. Effective prompt engineering minimizes ambiguous or incorrect answers (Zamfirescu-Pereira et al., 2023; Patil et al., 2023). (b) Rule-based customization—Setting predefined rules and response guidelines to complement the AI’s generative capabilities. This helps maintain consistency and prevents misleading or irrelevant responses (Rajala et al., 2023; Alessa and Al-Khalifa, 2023). (c) Fine-tuning with domain-specific data—If applicable, the chatbot can be trained on industry-specific datasets (e.g., healthcare, finance, education) to improve contextual understanding and response quality (Gill et al., 2024; Ciampa et al., 2024). (d) Identifying and addressing AI limitations—Recognizing areas where the chatbot struggles, such as handling highly complex queries or ethical concerns, and making necessary adjustments to improve performance (Pack and Maloney, 2023; Kim, 2023). This iterative training ensures that the chatbot is well-prepared for real-world interactions and aligns with its intended objectives (Gallo et al., 2023).

##### User testing and feedback refinement

Once the chatbot has been trained, it must be tested with real users to evaluate its effectiveness. This testing phase allows designers to identify strengths, weaknesses, and areas for improvement before full deployment. The process includes: (a) Beta testing with target users—Inviting a select group of users to interact with the chatbot and provide feedback on usability, accuracy, and overall experience (Ambikairajah et al., 2024; Limna et al., 2023). (b) Analyzing chatbot responses—Reviewing conversations to detect errors, inconsistencies, or instances where the chatbot fails to provide meaningful responses (Ekvall and Winnberg, 2023). (c) Iterative refinement—Adjusting chatbot settings, updating prompts, and modifying conversation flows based on user feedback to enhance functionality and engagement (Thapa and Patel, 2024). (d) Performance metrics evaluation—Tracking key metrics such as response time, user satisfaction, error rate, and completion rate to assess the chatbot’s effectiveness (Cerny, 2023; Heidari et al., 2024). By refining the chatbot through real-world interactions, developers can improve their reliability and responsiveness before official deployment.

##### Deployment and continuous monitoring

After successful testing and refinement, the chatbot is ready for deployment. However, launching the chatbot is not the final step—continuous monitoring and iteration are essential for long-term success. Deployment involves: (a) Launching the chatbot on designated platforms—Deploying the chatbot across websites, mobile apps, customer service portals, or messaging platforms based on the intended use case (Martinez-Araneda et al., 2023; Wang X. et al., 2024). (b) Real-world performance monitoring—Tracking chatbot interactions, analyzing user engagement patterns, and identifying recurring issues (Islam et al., 2024). (c) Automated and manual oversight—Implementing monitoring tools to detect chatbot malfunctions or unintended behavior while allowing developers to make manual adjustments when needed (Kim et al., 2024). (d) Ongoing iteration and updates—Using real-time data and feedback to refine the chatbot, introduce new features, or adjust existing functionalities for improved performance (Florindi et al., 2024). This framework allows for continuous improvements, ensuring that the chatbot evolves alongside user needs and technological advancements.

Phase 3 is crucial in ensuring that the chatbot operates efficiently, provides valuable interactions, and maintains high performance after deployment. By prioritizing training, testing, and iterative refinements, developers can create an adaptive and intelligent chatbot that meets user expectations. Continuous monitoring and updates will further enhance its long-term effectiveness, ensuring it remains a reliable tool for its intended applications (Limna et al., 2023; Patil et al., 2023).

### Case studies

Four articles were selected to demonstrate how the proposed framework performs and functions as well as demonstrating the versatility of the framework within chatbots utilizing different methodologies. Table 6 presents more information on these four articles as different case studies.

**Table 6:** Four case studies demonstrating the framework for designing effective chatbots.

	Case study 1 (Florindi et al., 2024)	Case study 2 (Gallo et al., 2023)	Case study 3 (Lee et al., 2024)	Case study 4 (Zhou, 2023)
Phase 1: ideation for chatbot				
Identify the problem	The article talks about challenges in optimizing chatbot interactions to enhance user engagement. Florindi et al., indicates intentions to improve the emotional component of chatbots for business applications. The main objective is improving the chatbot’s ability to understand user intent correctly and give relevant responses.	The article discusses the challenge of refining chatbot intellect to better comprehend user intent and provide more precise responses. The main issue addressed is improving chatbot adaptability and its performance in responding to a diverse range of queries.	Lee et al. (2024) indicates challenges of designing conversational agents that can provide personalized and adaptive healthcare support. The main problem is the lack of systems that combine contextual understanding, personalization, and effective interaction in healthcare atmospheres, especially when handling diverse user intents and emotions.	Zhou (2023) discusses the need to improve the intelligence and adaptability of teaching robots. The main objective is incorporating natural and effective human-machine communication into educational atmospheres using ChatGPT to overall improve support personalized education.
Target audience	The target users are business and professionals searching to integrate chatbots into their customer support and engagement techniques. Specifically focusing on companies trying to leverage AI-driven interactions to improve user experience.	The target audience of the article is primarily researchers, developers, and practitioners interested in conversational agents, smart environments (such as smart homes), and end-user development.	The target audience includes patients and individuals seeking healthcare information or support with digital systems. It also includes healthcare providers and developers of medical chatbot systems who aim to implement adaptive dialogue systems that respond meaningfully to user emotions and intents.	The target audience are educational professionals that include teachers and students at K-12 and higher education institutions. The article focuses on individuals and organizations that could be interested in intelligent teaching robots and the integration of conversational AI in the classroom.
Analytic tools used	Florindi et al. (2024) utilize tools such as Google Analytics, AI-driven customer insights platforms, and heatmaps to analyze user interactions and chatbot effectiveness.	The chatbot design process uses a variety of analytic tools to allow natural and effective interactions. Rasa is employed for its ability to perceive user intents, extract key pieces of information, and arrange conversational flows. ChatGPT is also used to handle more complex natural language processing tasks. Cosine similarity on text embeddings allows the chatbot to retrieve and utilize relevant information from a knowledge base, enhancing the accuracy and relevance of its responses.	The tools mentioned consisted of Natural Language Understanding (NLU) tools, speech recognition APIs, BERT for semantic similarity and context, dialogue act classifiers, and machine learning classifiers for emotion and intent recognition.	The article used ChatGPT as its main tool. Zhou highlights the use of AI-driven NLP and generation capabilities to replicate teaching interactions and student responses.
Analyzing the data	The article discusses methods like A/B testing, user feedback analysis, and engagement tracking to evaluate chatbot performance. Key metrics included user retention rates, chatbot accuracy, and session duration.	Gallo et al. explores the usage of large-scale datasets, performance standards to find out the chatbot capabilities, and sentiment analysis. Key metrics included contextual relevance, response coherence, and error reduction rates.	The study uses a multimodal dataset from user interactions. Annotations were created for emotions, dialogue acts, and intents, the data was then analyzed using machine learning models to evaluate accuracy in recognizing user needs. Evaluation metrics included F1-score, accuracy, and confused matrices.	The article explains the usage of test scenarios and feedback from simulated interactions between users and the teaching robot.
User persona	The article illustrates user persona including tech-savvy customers, business professionals seeking automation, and customer service teams relying on AI-assisted support. Their main goals are efficiency, ease of use, and quick issue solving.	The article showcases user personas such as AI developers fine-tuning chatbot models, researchers conducting experiments on language models, and users testing chatbot accuracy in controlled settings. Their main concerns include accuracy, relevancy, and decreasing biases in AI responses.	Lee et al. describes users as healthcare seekers who may be emotionally vulnerable, confused, or in need of reassurance. These personas typically expect quick access to medical support, accurate and emotionally aware responses, and interaction that feels like a human and context sensitive.	The article indicates key user personas as students seeking personalized help and teachers looking for interactive assistants in classroom environments. These users are personalized by a need for natural, logical communication and expect context aware, accurate responses from the robot.
Phase 2: chatbot design and development				
Feature integration	The chatbot uses features like intent recognition, multi-turn conversations, personalized responses based on user history, and contextual memory.	The chatbot system integrates advanced neural network-based understanding, multiturn conversation tracking to enhance user interactions, and self-learning capabilities.	Lee et al. indicates features integrated systems such as intent recognition, dialogue act classification, emotion detection, multimodal input handling, and contextual dialogue management.	The system incorporates ChatGPT’s capabilities including intent recognition, semantic understanding, reasoning capabilities, context memory, and multi-turn conversation.
Chatbot interaction	The chatbot is designed to greet users, handle FAQs, resolve customer complaints, and provide tailored recommendations based on previous interactions.	The chatbot in the article is designed to conduct deep conversational analysis, dynamically adjust responses based on context, and handle ambiguous user inputs with enhanced clarification systems	The chatbot handles diverse user intents like asking for medical advice, seeking clarification, and expressing anxiety. It tailors its responses by identifying the emotional tone and dialogue act, enabling for a more empathetic and accurate reply.	The chatbot is designed to engage in Q&A style conversations wiht user, replicate human-like teaching responses, and assist in problem-solving. It also provides explanations, correct misunderstandings, and guide students through educational content.
User friendly conversation paths	Florindi et al. (2024) mentions structuring conversation flows using interactive buttons, guided responses, and NLP for free text inputs.	Gallo et al. (2023) mentions strategies for refining chatbot interaction flows through hierarchical intent classification, improved dialogue modeling, and real time contextual adaptations.	The system is designed with modular dialogue paths. These are enhanced using dialogue act sequences, emotion driven response adjustment, and intent models.	Conversation flows are structured around human-computer interaction design principles. The system uses free-text NLP inputs to generate responses, focusing on natural and fluid conversations. It also adapts to the logic and language style of the user to maintain engagement.
Generative AI integration	The chatbot uses GPT based architectures.	The chatbot uses ChatGPT and RASA fine-tuned on domain specific datasets. It engages reinforcement learning with human feedback to improve conversational fluency and minimize errors in automated responses.	The chatbot integrates pretrained language models like BERT for semantic similarity calculations, context preservation, and emotion and intent classification. Training the Chatbot- Lee et al. mentioned the chatbot is trained using a custom labeled dataset	The chatbot uses generative AI such as ChatGPT. The model uses massive pre-trained language data. It is optimized by reinforcement learning with human feedback (RLHF) and supervised fine-tuning.
Phase 3: chatbot testing and deployment				
Training the chatbot	Training was done using machine learning models, supervised learning, and reinforcement learning. The chatbot improves by analyzing customer queries, feedback loops, and predefined datasets.	The chatbot is trained using supervised learning, continuous feedback loops, and reinforcement learning. Training data includes diverse conversational datasets, error correction systems, and user feedback logs.	The chatbot integrates pretrained language models like BERT for semantic similarity calculations, context preservation, and emotion and intent classification. Training the Chatbot- Lee et al. mentioned the chatbot is trained using a custom labeled dataset combining intent, motion and dialogue act annotations. Supervised machine learning methods and tools like Random Forest, CGBoost, and deep learning models were also utilized.	Training is based on the GPT-3.5 architecture using large scale language collection. Specific datasets are not mentioned. However, the system benefits from reinforcement learning and supervised fine-tuning.
Testing the chatbot	The article mentions QA testing, beta testing with selected users, and real time deployment in controlled environments for iterative improvements.	Gallo et al. (2023) describes testing methods including real-world user simulation, automated response validation, and adversarial testing to ensure chatbot reliability.	Methods used for testing included offline evaluations, model comparisons, manual annotation validation, and multimodal analysis.	Zhou mentions testing through designed interactive sessions and scenario-based question-answering tasks. These are used to evaluate accuracy, logic, fluency, and semantic relevance in responses. Feedback loops are used to optimize the conversation generation process.
Chatbot deployment	The chatbot is deployed via cloud-based services and integrated into websites, messaging apps, and mobile platforms. The article details strategies for monitoring performance and scaling based on demand.	The chatbot is deployed using scalable cloud infrastructure, integrated into web and mobile platforms, and continually monitored for performance enhancements through automated logging and periodic updates.	The chatbot is still in the research and development phase. However, the article implies a future deployment vision involving cloud-based architecture and integration with healthcare platforms. Deployment considerations include scalability, real-time processing, and privacy-preserving data handling.	The chatbot is integrated into the teaching robot system and can be deployed by cloud-based services, mobile platforms, or desktop software. Zhou discusses future plans for broader educational deployment and performance optimization through user interaction feedback.

#### Case study 1: a novel solution for the development of a sentimental analysis chatbot integrating ChatGPT

This chatbot improves emotional understanding in conversations by using sentiment detection and contextual memory to adjust responses based on user mood. In the Ideation phase, the study identifies challenges in optimizing chatbot interactions to improve user engagement, particularly in business applications. The Design and Development phase focuses on features like intent recognition, multi-turn conversations, and personalized responses, using GPT-based architectures. The Testing and Deployment phase involves QA testing, beta testing with selected users, and real-time deployment in controlled environments, with deployment strategies emphasizing performance monitoring and scalability via cloud services and integration into various platforms. The benefit is in its ability to improve customer satisfaction, automate emotional support, and boost engagement by offering responses that feel personally tailored.

#### Case study 2: toward a chatbot for creating trigger-action rules based on ChatGPT and Rasa

This chatbot leverages transformer models and reinforcement learning to understand user goals and generate multi-turn, context-aware conversations. During Ideation, the study addresses refining chatbot intellect to better understand user intent and provide more precise responses within smart environments. Design and Development integrates advanced neural network-based understanding, multi-turn conversation tracking, and self-learning capabilities, fine-tuning ChatGPT and RASA on domain-specific datasets, and reinforcement learning. Testing and Deployment includes real-world user simulation, automated response validation, and adversarial testing to ensure chatbot reliability. The deployment is executed using scalable cloud infrastructure, integrated into web and mobile platforms, with continual monitoring and automated logging for performance enhancements.

#### Case study 3: development of AI–generated medical responses using the ChatGPT for cancer patients (Lee et al., 2024)

This medical chatbot combines emotion recognition, intent classification, and dialogue act analysis to support cancer patients with empathetic and informative conversations. For Ideation, the study focuses on designing conversational agents that can provide personalized and adaptive healthcare support. The Design and Development phase integrates features such as intent recognition, emotion detection, multimodal input handling, and contextual dialogue management using pre-trained language models like BERT. The Testing phase includes offline evaluations, model comparisons, manual annotation validation, and multimodal analysis. While the chatbot is in research and development, future deployment visions involve cloud-based architecture and integration with healthcare platforms, emphasizing scalability and privacy.

#### Case study 4: application research of teaching robot based on ChatGPT

Integrated into a teaching robot, this chatbot uses ChatGPT to deliver personalized, logically structured educational support. The Ideation phase discusses improving the intelligence and adaptability of teaching robots to enhance personalized education. Design and Development incorporates ChatGPT’s capabilities, including intent recognition, semantic understanding, reasoning, and context memory. The chatbot is trained using large-scale language data and optimized with reinforcement learning and supervised fine-tuning. Testing involves designed interactive sessions and scenario-based question-answering tasks. Deployment includes integration into teaching robot systems via cloud services, mobile platforms, or desktop software, with plans for broader educational deployment and performance optimization through user interaction feedback.

### Advanced technical framework for ChatGPT chatbots: procedures and implementation

This expanded section outlines the precise steps and rationale for building, integrating, fine-tuning, and evaluating ChatGPT-based chatbots, with a focus on current best practices, peer-reviewed methods, and direct implementation details. All statements are supported by rigorously selected journal sources.

#### Data preprocessing pipeline

Data preprocessing is foundational to chatbot performance; it transforms raw, heterogeneous conversational inputs into a structured, model-friendly format. The procedure involves automatic filtering, normalization, labeling, and rigorous validation.

##### Step 1: data acquisition and initial cleansing

Initiate by compiling large-scale, transactional or support datasets (e.g., customer logs, forum transcripts, annotated dialog pairs) following ethical data acquisition guidelines. Automated scripts should remove obvious incomplete sessions, spam, and non-task activities, reducing manual review time.

##### Step 2: advanced cleaning and feature extraction

Use AI-powered algorithms (such as recursive feature elimination or tree-based model ranking) to identify and retain the most salient features for conversational modeling (Sivathapandi et al., 2022). Apply deep learning-based noise detection to remove outliers and irrelevant entries—enhancing both accuracy and interpretability.

##### Step 3: annotation and labeling

Employ trained annotators and semi-automatic tools to tag intents, entities, sentiment, escalation triggers, and special user needs. For scalability, incorporate active learning approaches where models suggest uncertain tags for human verification, optimizing annotation resources.

##### Step 4: data normalization and augmentation

Leverage normalization tools (tokenization, lemmatization, lowercasing) to standardize flow, while employing paraphrasing and back-translation for augmentation, especially when minority classes (rare intents) are underrepresented. Use dimensionality reduction (PCA, t-SNE) for high-dimensional input, preparing datasets for transformer models.

##### Step 5: validation, split, and quality control

Perform cross-validation checks, class balancing, and session consistency testing. Data is then split (commonly 70% train, 15% validation, 15% test) with stratified sampling to ensure minority classes are adequately represented in all splits. Quality is further confirmed by automated and manual audits following best practices for conversational dataset preparation.

#### Model integration workflow

The integration workflow transforms preprocessed data into actionable, context-aware chatbot interactions via transformer-based models.

##### Step 1: API configuration and security

Implement robust authentication for external API calls (secure storage of keys, encrypted transactions). Configure endpoint parameters including model version, temperature, and output length according to domain needs.

##### Step 2: custom prompt engineering

Design advanced prompt templates integrating context history, user metadata, and domain constraints to maximize response relevance (Wang H. et al., 2024). This includes rolling windows of user input to maintain session continuity and allow adaptive response generation.

##### Step 3: conversation state management

Use session IDs and context windows to pass prior conversation turns into each new API call, enabling the transformer to maintain logic and narrative flow. Leverage intent recognition modules and relevance scoring for context prioritization.

##### Step 4: response parsing and routing

Output from the LLM must be parsed for actionable elements (commands, recommendations, user escalation triggers). Responses are formatted for UI integration (HTML, markdown), and filtered for compliance with privacy and ethical standards.

##### Step 5: failover and fallback handling

Implement robust fallback procedures for empty or toxic outputs; for example, if a response is null or violates safety constraints, switch to pre-scripted templates or escalate to a human operator.

#### Fine-tuning procedures

Domain adaptation through supervised fine-tuning is the key to expert-level response quality.

##### Step 1: dataset construction and ethics review

Prepare a labeled dataset capturing domain-specific knowledge (e.g., healthcare Q&A, troubleshooting logs), ensuring privacy, diversity, and compliance (Balavadhani et al., 2024). Include bilingual examples if multi-language support is required.

##### Step 2: model selection and hyperparameter tuning

Select a transformer architecture suitable for the application scale (GPT-3.5, GPT-4, Llama v2). Use grid-search or Bayesian optimization to determine the optimal hyperparameters—learning rate, batch size, epochs, context window.

##### Step 3: supervised fine-tuning

Apply supervised learning on the labeled dataset, tracking key metrics (perplexity, BLEU, accuracy) to ensure overfitting is avoided. Use frameworks like Huggingface Trainer or OpenAI fine-tuning API for reproducibility.

##### Step 4: reinforcement learning from human feedback

Iteratively improve response quality by integrating user feedback as a reward function for RL-based tuning, optimizing for relevance, politeness, and factual correctness (Zhou et al., 2023).

##### Step 5: deployment and monitoring

Deploy fine-tuned models using container orchestration (Docker, Kubernetes) to ensure scalability. Monitor real-time performance, log interaction outcomes, and schedule periodic retraining as new user data is collected.

#### Performance monitoring architecture

Ongoing monitoring enables reliability, scalability, and iterative improvement.

##### Step 1: real-time analytics dashboard

Integrate dashboards (Grafana, Kibana) connected to a structured logging backend (Elasticsearch, MongoDB), providing up-to-the-minute metrics for query latency, throughput, error rates, and user satisfaction (Motger et al., 2022).

##### Step 2: multi-level evaluation metrics

Use precision, recall, F1-score, human-likeness metrics (BLEU, ROUGE, METEOR) and natural language understanding benchmarks to evaluate and compare system performance per release cycle. Automated evaluation metrics are critical for assessing chatbot performance. Logeswaran and Lee (2018) developed fluency metrics specifically designed for chatbot interactions, while Huang (2021) established foundational frameworks for response assessment.

##### Step 3: user feedback integration

Automate collection of CSAT and NPS ratings post-interaction, analyze abandonment rates, and flag negative user sentiment for quality reviews.

##### Step 4: anomaly and security event detection

Deploy intrusion detection (IDS) and anomaly monitoring for outlier response patterns, toxic language, and abnormal traffic, followed by automated alerts and escalation (Perez et al., 2022).

##### Step 5: continuous improvement loop

Schedule regular review cycles, retrain models with newly collected feedback, and update prompt templates based on recent failure modes to maintain and advance chatbot quality.

## Discussion and conclusion

This systematic literature review highlights the critical elements of designing intelligent chatbots with ChatGPT by addressing three core thematic questions: user experience (UX) and engagement, hybrid design models, and the influence of prompts on chatbot performance. These interconnected themes offer valuable insights into both research and practical applications, emphasizing the importance of balancing technical innovation with user-centric design principles. Studies emphasized the significance of UX and user engagement in shaping chatbot effectiveness. User satisfaction and interaction quality were closely linked to well-crafted chatbot experiences, with studies demonstrating how tools like the PromptBoard facilitate creative design and user interaction (Ekvall and Winnberg, 2023). The findings suggest that effective UX design enhances user satisfaction but highlights challenges such as maintaining creative ownership and managing repetitive AI responses. In educational contexts, tailored prompts significantly influenced user engagement by ensuring age-appropriate, culturally sensitive interactions (Koyuturk et al., 2023). These insights underscore the necessity of prompt engineering to align chatbot behavior with diverse user needs. Beyond summarizing the literature, this discussion synthesizes patterns across case studies, showcasing why certain hybrid design approaches (e.g., combining retrieval-augmented generation with domain-specific fine-tuning) consistently excel above others in areas such as healthcare and education. These advantages usually occur from improved fact-based support, reduced hallucinations, and higher field relevance. Although, critical fields also revealed limitations, especially in safety-sensitive situations where ChatGPT generated responses reliable references or shown ethical weaknesses.

The review of nine studies on hybrid design models reveals the advantages of integrating multiple frameworks to overcome the limitations of single model chatbots. Hybrid models, such as the combination of ChatGPT with Rasa or EmoRoBERTa, demonstrated superior performance in context retention, emotional intelligence, and scalability (Gallo et al., 2023; Florindi et al., 2024). By distributing tasks across generative and rule-based systems, hybrid designs achieved better accuracy and reliability, particularly in specialized domains like smart home management and customer service. Ethical considerations, including fairness, transparency, and bias mitigation, were often recognized but rarely implemented in practice. Several studies indicated risks of over-reliance on LLM outputs without reliable human guidance, especially in legal, medical, and financial applications. Addressing these challenges requires both technical safeguards and management policies. These findings suggest that future research should explore optimizing hybrid designs for scalability and user adaptability.

Fourteen studies focused on the challenges of prompt engineering, highlighting how prompt sensitivity affects chatbot reliability. Alessa and Al-Khalifa (2023) demonstrated that medium-detail prompts provide a balance between personalization and efficiency, while Zamfirescu-Pereira et al. (2023) noted the difficulty of achieving consistent responses due to prompt fickleness. The need for iterative testing and refinement of prompts emerged as a key finding, indicating that prompt-based systems are better suited for low-stakes applications unless integrated with other methodologies for greater control.

This review provides a comprehensive framework for designing ChatGPT-based chatbots, emphasizing the interplay between user experience, hybrid design, and prompt engineering. For researchers, it opens avenues for investigating adaptive prompt strategies and refining hybrid models to enhance chatbot flexibility and accuracy. Practitioners are advised to prioritize prompt customization and hybrid integration to meet specific user needs while addressing the inherent limitations of single-model designs. By bridging the gap between technical capability and practical application, this framework equips developers, researchers, and business leaders with actionable insights to create more intelligent, user-centric chatbot solutions.

## Data Availability

The data presented in this study can be accessed online. These details can be found in the article/Supplementary material.
